# *Bacillus* integrative plasmid system combining a synthetic gene circuit for efficient genetic modifications of undomesticated *Bacillus* strains

**DOI:** 10.1186/s12934-022-01989-w

**Published:** 2022-12-14

**Authors:** Man Su Kim, Da-Eun Jeong, Soo-Keun Choi

**Affiliations:** 1 Infectious Disease Research Center, Korea Research Institute of Bioscience and Biotechnology (KRIBB), Daejeon, 34141 South Korea; 2 Department of Biosystems and Bioengineering, KRIBB School of Biotechnology, University of Science and Technology (UST), Daejeon, 34113 South Korea

**Keywords:** *Bacillus* integrative plasmid, Synthetic gene circuit, Undomesticated *Bacillus*, Genetic modification, Integrative and conjugative element

## Abstract

**Background:**

Owing to CRISPR-Cas9 and derivative technologies, genetic studies on microorganisms have dramatically increased. However, the CRISPR-Cas9 system is still difficult to utilize in many wild-type *Bacillus* strains owing to Cas9 toxicity. Moreover, less toxic systems, such as cytosine base editors, generate unwanted off-target mutations that can interfere with the genetic studies of wild-type strains. Therefore, a convenient alternative system is required for genetic studies and genome engineering of wild-type *Bacillus* strains. Because wild-type *Bacillus* strains have poor transformation efficiencies, the new system should be based on broad-host-range plasmid-delivery systems.

**Results:**

Here, we developed a *Bacillus* integrative plasmid system in which plasmids without the replication initiator protein gene (*rep*) of *Bacillus* are replicated in a donor *Bacillus* strain by Rep proteins provided in trans but not in *Bacillus* recipients. The plasmids were transferred to recipients through a modified integrative and conjugative element, which is a wide host range plasmid-delivery system. Genetic mutations were generated in recipients through homologous recombination between the transferred plasmid and the genome. The system was improved by adding a synthetic gene circuit for efficient screening of the desired mutations by double crossover recombination in recipient strains. The improved system exhibited a mutation efficiency of the target gene of approximately 100% in the tested wild-type *Bacillus* strains.

**Conclusion:**

The *Bacillus* integrative plasmid system developed in this study can generate target mutations with high efficiency when combined with a synthetic gene circuit in wild-type *Bacillus* strains. The system is free of toxicity and unwanted off-target mutations as it generates the desired mutations by traditional double crossover recombination. Therefore, our system could be a powerful tool for genetic studies and genome editing of Cas9-sensitive wild-type *Bacillus* strains.

**Supplementary Information:**

The online version contains supplementary material available at 10.1186/s12934-022-01989-w.

## Background

*Bacillus* species are the most important commercial microorganisms used for the production of industrial enzymes, secondary metabolites, agricultural agents, and food fermentation [1]. The genus *Bacillus* is a large group with over 280 species [2], and new species are continually being discovered in new environments, such as the deep sea and human gut [3–5]. Wild-type *Bacillus* strains can grow under a wide range of conditions, such as salinity, pH, and temperature, and produce a variety of enzymes and chemicals. Therefore, they have great advantages as cell factories for the production of industrial and pharmaceutical proteins and chemicals [6]. Recent extensive genome analysis has revealed that *Bacillus* species are a rich source of novel antibiotics [7]. However, genetic studies on the development of cell factories and metabolic engineering have focused on well-characterized strains, particularly domesticated *Bacillus subtilis* 168. Most wild-type strains remain unexplored for commercial application because of the lack of suitable genetic tools.

Genetic modification of *Bacillus* strains has traditionally been accomplished through the integration of selective markers into target genes by double crossover recombination. Counter-selectable markers have been also used for marker-free genome editing in *B. subtilis* [8, 9]. Clustered regularly interspaced short palindromic repeat (CRISPR)-Cas9, a powerful and highly specific gene-editing system, and its derivatives have recently become mainstream genetic research [10]. These systems have mainly been used for genome editing of *B. subtilis* strains [11–16]. Several wild-type strains other than *B. subtilis*, such as *Bacillus amyloliquefaciens* [17], *Bacillus licheniformis* [18], *Bacillus thuringiensis* [19], *Bacillus anthracis* [20], and *Bacillus cereus* [20] have also been engineered using these systems. However, applying the CRISPR-Cas9 system to most wild-type strains is challenging owing to Cas9-related toxicity. Moreover, Cas9 expression is highly toxic in many microbes [21, 22]. Recently, a method using a cytosine base editor (CBE), which is less toxic than the Cas9 system, has been successfully used for multiplex genome editing of various wild-type *Bacillus* strains [23]. However, CBE induces off-target mutations in the genome, indicating that it cannot be used in genetic studies for gene regulation and functional analysis. Therefore, a convenient method without toxicity or off-target mutations is required for genetic studies of wild-type *Bacillus* strains.

Traditional plasmids called “integrative plasmids” for genetic modifications of wild-type *Bacillus* strains contain an integration cassette for the modifications of *Bacillus* recipients and origin of replication for *Escherichia coli* but not for *Bacillus*. Integrative plasmids are suicide vectors that can replicate in *E. coli* but not in *Bacillus* recipients. Upon transferring the plasmids purified from *E. coli* to *Bacillus* recipient cells, only transformants in which the cassette is integrated into the chromosome can grow in a selective medium. This integration creates a knockout mutation in the target gene. Electroporation or conjugation from an *E. coli* donor to a *Bacillus* recipient has been used for the transformation of wild-type *Bacillus* strains. However, many wild-type strains cannot be transformed using either method [24]. Therefore, an efficient method for plasmid delivery with a wide host range is required to genetically modify wild-type *Bacillus* strains. Recently, an integrative and conjugative element (ICE)-based DNA transfer method has demonstrated wide host-range characteristics [25]. In addition, a modified ICE (MICE) capable of delivering plasmids from *Bacillus* donors to a wide range of undomesticated *Bacillus* strains has been reported [24]. However, in the MICE system, both the donor and recipient are *Bacillus* cells. This indicates that the plasmids can be replicated in both donors and recipients, making it difficult to induce genetic mutations in the recipient genome by double crossover recombination. Therefore, in this study, we developed a *Bacillus* integrative plasmid (BIP) system in which the plasmid can replicate in a *Bacillus* donor but not in a *Bacillus* recipient. The plasmid can be transferred via MICE to induce genetic mutations in the recipient cells. We also improved the system by adding a synthetic gene circuit to enhance the screening of mutations in recipients. This system may be useful for generating genetic mutations in wild-type *Bacillus* strains that cannot be achieved using CRISPR-Cas9 or derivative techniques.

## Results

### Construction of a BIP system

**Fig. 1:**
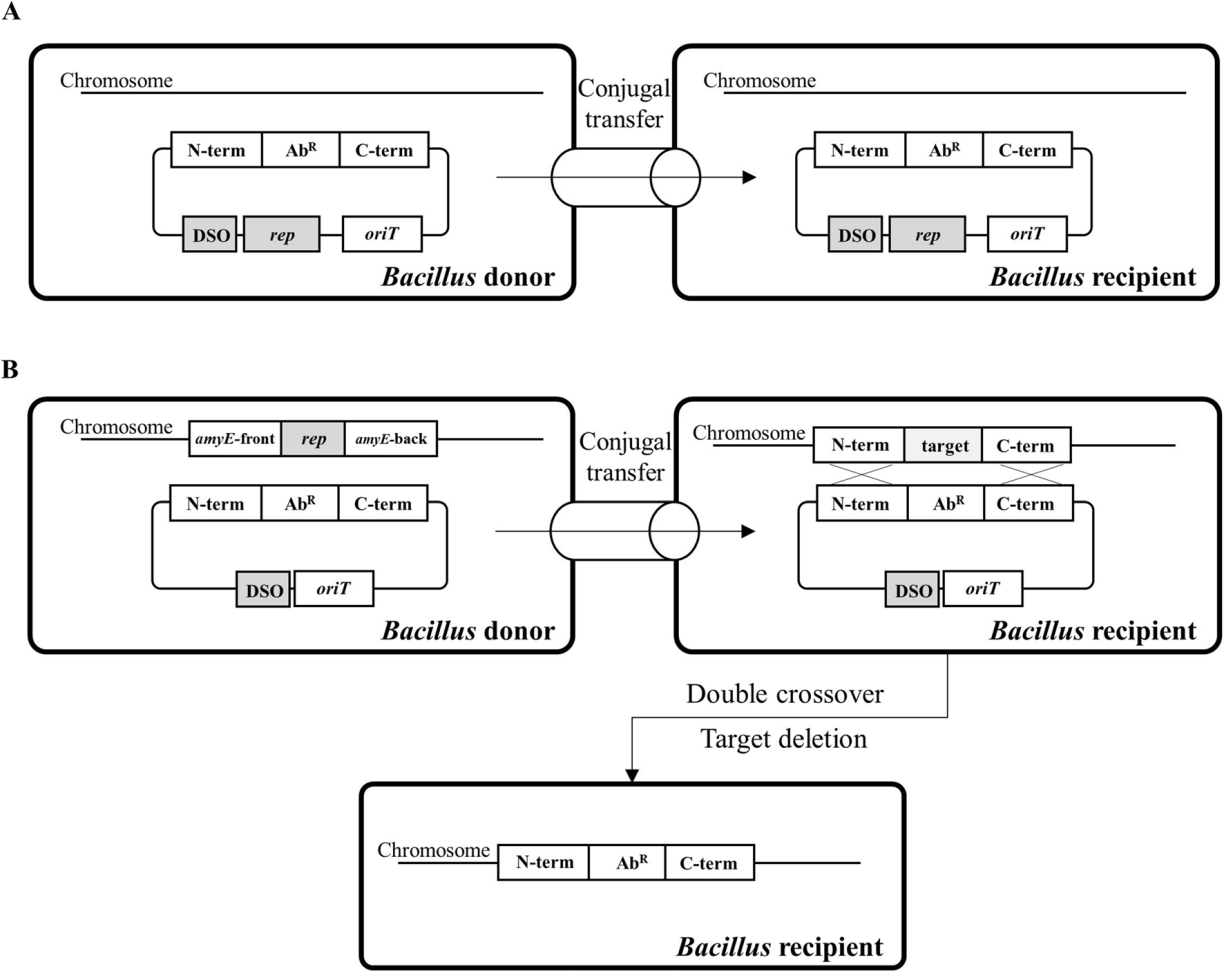
Scheme of plasmid transfer by conjugation. **A** Conjugal transfer of general plasmids from a *Bacillus* donor to a *Bacillus* recipient. The plasmid contains a replication initiator gene (*rep*) and its cognate double-strand origin of replication (DSO), allowing it to exit in plasmid form in both donor and recipient cells. **B** Conjugal transfer of integrative plasmids from a *Bacillus* donor to a *Bacillus* recipient. The integrative plasmid contains a DSO but not *rep*. Thus, it can be replicated in donor *Bacillus* cells by providing Rep in trans, but not in recipient *Bacillus* cells

**Fig. 2:**
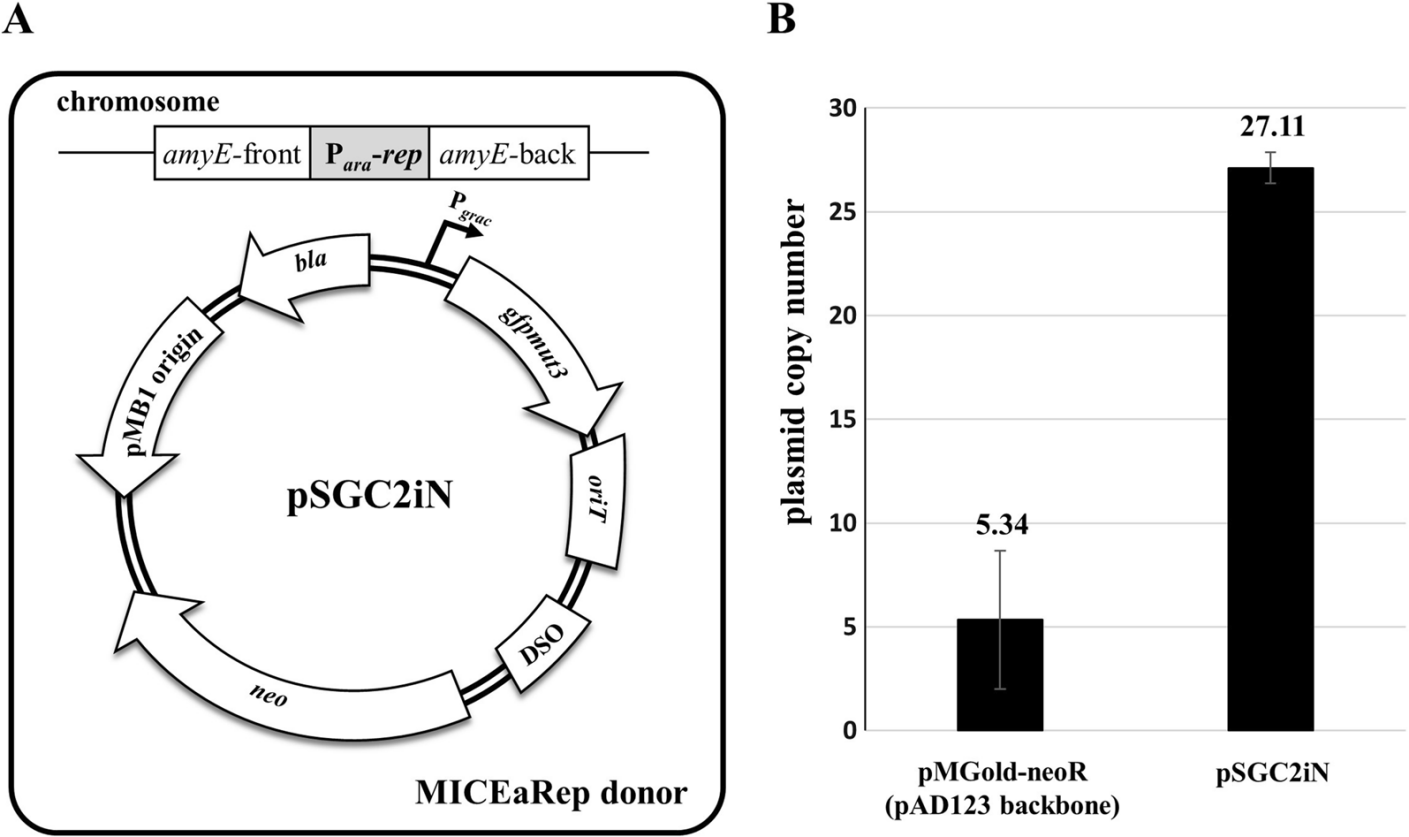
Construction of integrative plasmid and conjugative donor strain for the *Bacillus* integrative plasmid (BIP) system. **A** Construction of the BIP system. The MICEaRep (a donor strain for modified integrative and conjugative element [MICE] system) was constructed by inserting the replication initiation gene (*rep*) of plasmid pAD123 into the *amyE* locus of the chromosome. The *rep* gene was under the control of the constitutive promoter P_*ara*_ which is modified from the *Bacillus subtilis ara* promoter. The BIP backbone, pSGC2iN, has a double-strand origin of replication (DSO) region and a pMB1 origin for *E. coli* but not the *rep* gene for *Bacillus*. **B** Quantitative real-time PCR to determine the plasmid copy number (PCN) for pAD123 backbone in *B. subtilis* 168 and pSGC2iN in MICEaRep donor. The *dnaN* and *neo* genes were selected as a single-copy reference gene on the chromosome and the target gene on the plasmid, respectively. The PCN was calculated via dividing the target plasmid absolute quantity by the chromosomal absolute quantity in the template total DNA. The bars display the means of three independent measurements, with the error bars indicating standard deviations

The plasmid pAD123 [26] containing the pTA1060 replication origin replicates by a rolling-circle mechanism with replication initiator proteins (Rep) and its cognate double-strand origin of replication (DSO) in *Bacillus* (Additional file 1: Fig. S1) [27]. In general, the pAD123 derivatives containing the origin of transfer (*oriT*) can be transferred to the recipient by conjugation (Fig. 1A). Here, we modified the plasmid pAD123 to construct a BIP system, where the plasmid still contained the DSO region but not the *rep* gene which was integrated into the chromosome of a *Bacillus* donor instead (Fig. 1B). Transformants were not obtained when the modified plasmid pSGC2iN lacking the *rep* gene (Fig. 2A) was introduced into the *B. subtilis* MICE donor BS5918 (Additional file 1: Table S1). Next, we introduced the plasmid pSGC2iN into *Bacillus* donor MICERep, which contains the *rep* gene of pAD123 in its chromosome. However, the transformants showed a markedly slow growth rate on LB plates containing a selective concentration of neomycin (10 µg/mL), and plasmids from the transformants could not be detected by agarose gel electrophoresis analysis. These results suggest that the expression level of the chromosomally integrated *rep* gene with the native promoter was not sufficient to maintain BIP. Therefore, we replaced the native promoter of *rep* in the chromosome of the MICERep strain with a constitutive promoter to construct the donor strain MICEaRep (Fig. 2A). When the pSGC2iN plasmid was transferred to MICEaRep, the growth rate of the transformants was normal, and the plasmids purified from the transformants were detected by agarose gel electrophoresis. The plasmid copy number of pMGold-neoR, a plasmid containing the *rep* gene in the pAD123 backbone, was 5.34, similar to that previously reported [26], whereas that of BIP in the MICEaRep donor was 27.11 (Fig. 2B).

### Construction of a BIP system combining a synthetic gene circuit

**Fig. 3:**
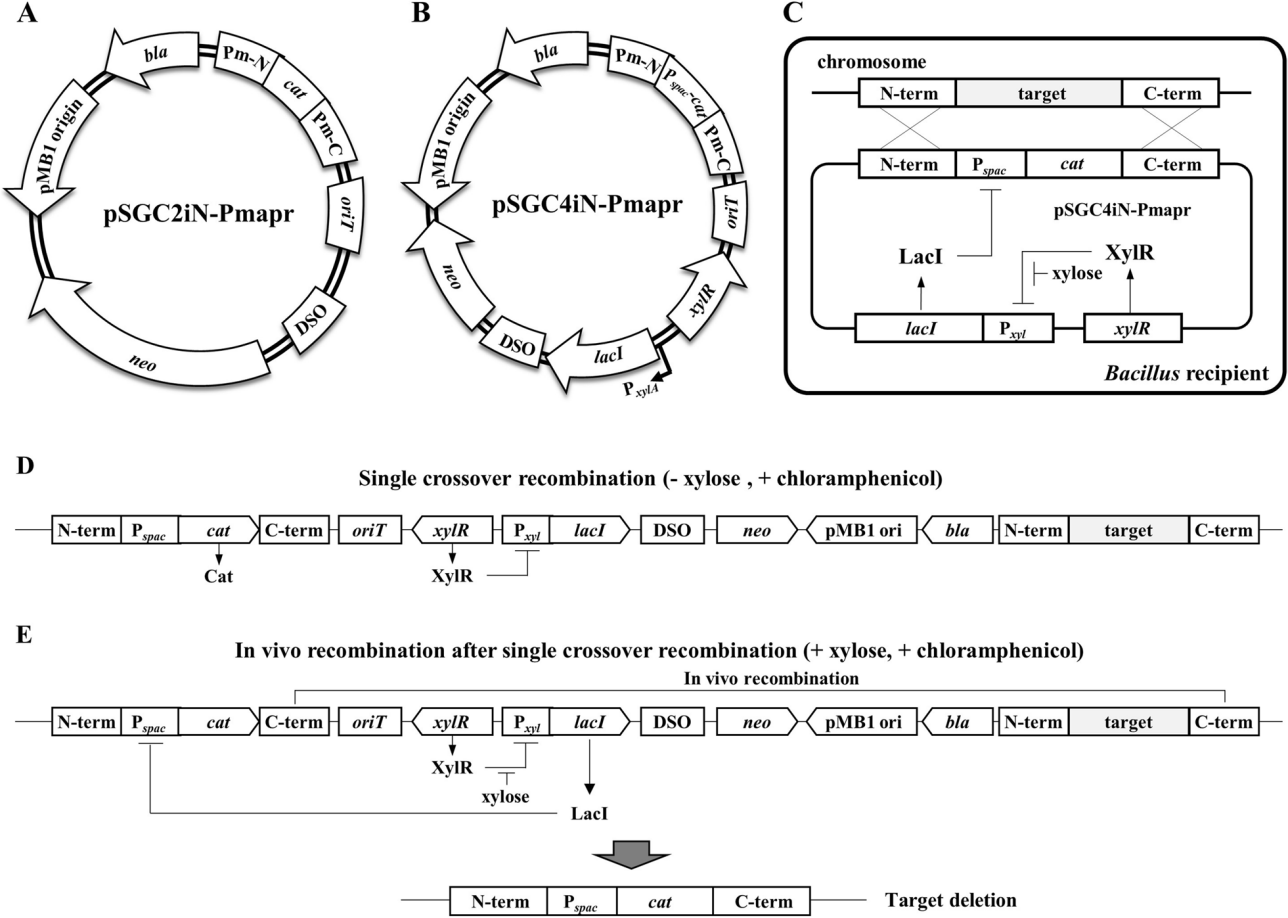
The construction of a *Bacillus* integrative plasmid (BIP) and a BIP combining a synthetic gene circuit (BIPS) to delete the *aprE* gene of *B. pumilus*. **A** The pSGC2iN-Pmapr for *B. pumilus aprE* gene deletion via the BIP system was constructed by cloning homologous arms (Pm-N and Pm-C) and chloramphenicol resistance gene (*cat*) to pSGC2iN. **B** The construction of pSGC4iN-Pmapr to delete the *aprE* gene of *B. pumilus* via the BIPS system. The synthetic gene circuit of pSGC4iN-Pmapr consists of *xylR*-P_*xylA*_-*lacI* and PmN-P_*spac*_-*cat*-PmC cassettes that enhance recipient mutation selection. **C** The scheme of the synthetic gene circuit to function as a counter-selectable marker. In the absence of xylose, the XylR repressor blocks *lacI* gene expression and the *cat* gene is expressed, rendering the recipient resistant to chloramphenicol. In contrast, the addition of xylose induces *lacI* expression, which suppresses *cat* gene expression, making cells sensitive to chloramphenicol. **D** Structure of target chromosome when BIPS is inserted through single crossover integration (SCO). Transformants with the entire plasmid integrated into the chromosome by SCO can survive in the absence of xylose and the presence of chloramphenicol. **E** Induction of in vivo recombination from SCO colonies. The survival of cells containing the entire plasmid in the presence of xylose and chloramphenicol requires the deletion of the P_*xyl*_-*lacI* cassette by in vivo recombination between homologous C-terminal fragments. In vivo recombination will generate genomic mutations

*Bacillus pumilus* was selected as the test strain to determine whether the BIP system could be transferred via MICE and induce genetic mutations in wild-type recipients. We constructed a plasmid, pSGC2iN-Pmapr, containing two homologous arms (Pm-N and Pm-C) flanking a chloramphenicol resistance gene (*cat*), to delete the *aprE* gene of *B. pumilus* via the BIP system (Fig. 3A). Because pSGC2iN-Pmapr does not have an origin of replication for *Bacillus*, only transformants with chromosomally integrated plasmids can be grown on selective agar plates. The entire plasmid may be inserted by a single crossover or only homologous arms may be inserted by a double crossover. The single crossover is resistant to both chloramphenicol and neomycin but does not induce mutagenesis, whereas the double crossover is resistant only to chloramphenicol and induces mutagenesis. Transformation of *B. pumilus* with pSGC2iN-Pmapr resulted in most transformants displaying both chloramphenicol- and neomycin-resistant phenotypes (Additional file 1: Fig. S2). PCR analysis confirmed that the transformants contained a single crossover integration of the plasmid into the chromosome. Although desired mutations can be obtained from transformants through negative selection by culturing in a non-selectable medium to induce in vivo recombination, it is a low-efficiency, labor-intensive, and time-consuming method. Therefore, we developed a BIP system that combines a synthetic genetic circuit (BIPS) capable of positive selection when screening for the desired mutations.

**Fig. 4:**
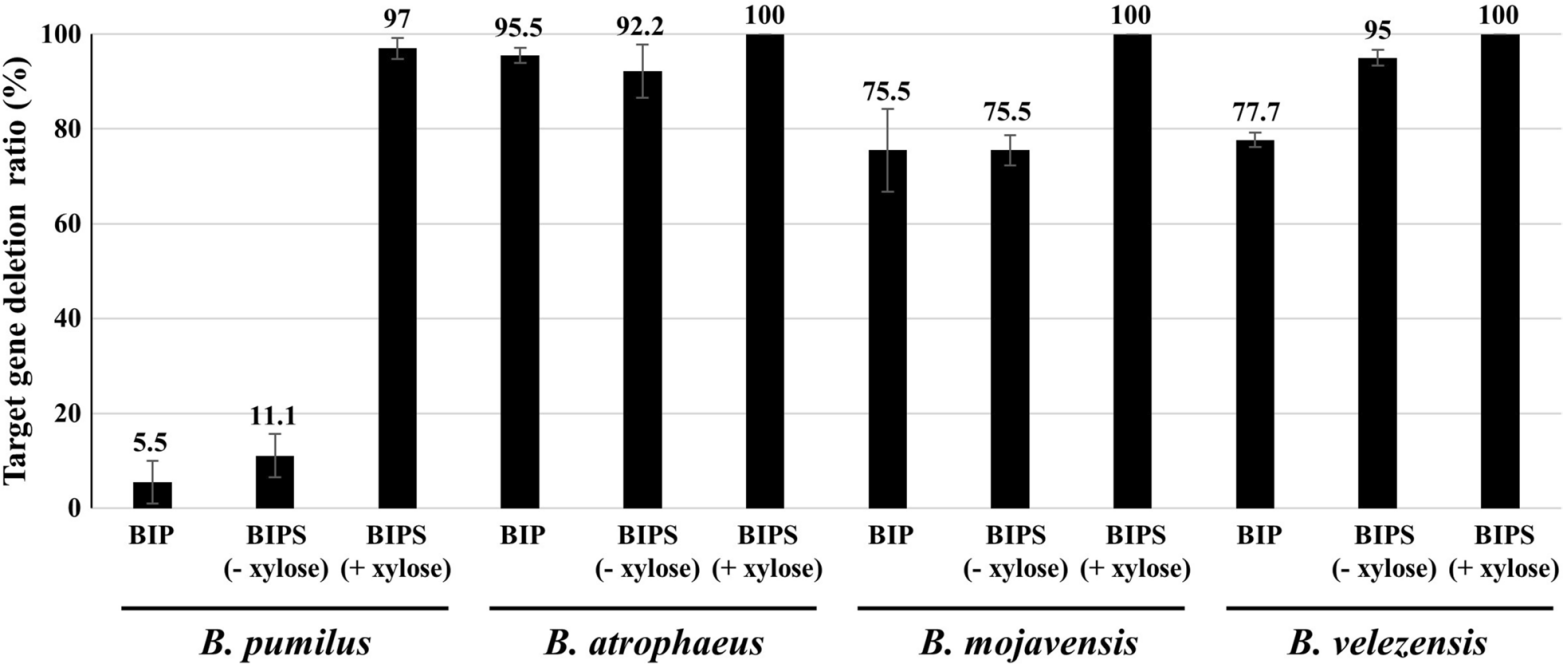
Target gene deletion ratios by BIP and BIPS systems for *B. pumlus*, *B. atrophaeus*, *B. mojavensis*, and *B. velezensis*. The ratio was calculated by dividing the number of colonies showing chloramphenicol resistance and neomycin sensitivity by the obtained colonies. The bars display the means of three independent measurements, with the error bars indicating standard deviations. BIP, *Bacillus* integrative plasmid; BIPS, BIP with synthetic gene circuit

The plasmid pSGC4iN-Pmapr for the BIPS system contains a *xylR*, a *cat* gene under the promoter P_*spac*_, and a *lacI* under the promoter P_*xylA*_. P_*spac*_-*cat* was flanked between the homologous arms (Fig. 3B). In the absence of xylose, the XylR repressor blocked *lacI* gene expression, and the *cat* gene was expressed, resulting in resistance to chloramphenicol (Fig. 3C). Thus, transformants with the entire plasmid integrated into the chromosome by single crossover recombination can survive in the absence of xylose and the presence of chloramphenicol (Fig. 3D). In contrast, the addition of xylose induced *lacI* expression, which suppressed *cat* gene expression, rendering the cells sensitive to chloramphenicol. Thus, the survival of cells containing the entire plasmid in the presence of xylose and chloramphenicol requires the deletion of the P_*xyl*_-*lacI* cassette by in vivo recombination between homologous C-terminal fragments. In vivo recombination generated genomic mutations (Fig. 3E). When BIP (pSGC2iN-Pmapr) was transferred to *B. pumilus*, single crossover recombination occurred in most transformants, and double crossover recombination occurred in only 5.5% of the transformants showing deletion of the target gene. When BIPS (pSGC4iN-Pmapr) was transferred to *B. pumilus*, the target deletion rate was 11.1% in the absence of xylose. However, culturing transformants with a single crossover integration of BIPS in the presence of xylose (1%) and 50 µg/mL chloramphenicol increased the target deletion rate to 97% (Fig. 4). Moreover, the target deletion was confirmed by PCR and sequencing (Additional file 1: Fig. S3A and S3B).

### Application of BIPS to various *Bacillus* species

The applicability of BIPS to *Bacillus* species other than *B. pumilus* was investigated by introducing BIPS into *Bacillus atrophaeus*, *Bacillus mojavensis*, and *Bacillus velezensis*. When BIP was introduced into *B. atrophaeus*, *B. mojavensis*, and *B. velezensis*, the target gene deletion rates were 95.5, 75.5, and 77.7%, respectively, which were higher than those in *B. pumilus* (Fig. 4). In the absence of xylose, the gene deletion rates with the BIPS system were 92.2, 75.5, and 95%, in *B. atrophaeus*, *B. mojavensis*, and *B. velezensis*, respectively. In the presence of xylose, the target deletion rate was 100% in all strains (Fig. 4). The target deletion was confirmed by PCR and sequencing (Additional file 1: Fig. S3A and S3B). These results suggest that BIPS can be used to effectively induce genomic mutations in various wild-type *Bacillus* strains.

## Discussion

The difficulty of transforming wild-type *Bacillus* strains needs to be overcome to realize the medical and industrial potential of various *Bacillus* strains. Although the CRISPR-Cas9 system enables genome engineering in diverse organisms [28], Zhao et al. reported that the Cas9 system is difficult to apply to wild-type strains owing to its toxicity and off-target effects, thereby limiting its utility for novel microorganisms [21]. In contrast, the BIP/BIPS systems developed here are non-toxic to cells and do not produce off-target mutations, allowing for them to be used for targeted mutagenesis in various wild-type *Bacillus* strains. Moreover, because MICE is a broad-host-range plasmid-delivery system, BIP/BIPS using the MICE system can be applied to engineer a wide range of wild-type *Bacillus* strains, including strains in which CRISPR-Cas9 is difficult to apply.

In the BIP/BIPS systems, the *rep* gene from the pTA1060 origin of replication was separated from the cognate DSO region and placed on the chromosome of the donor strain. BIP/BIPS plasmids function as suicide vectors capable of replicating in *Bacillus* donors but not recipients. If a *Bacillus* recipient has a *rep* gene that is highly homologous to that of pTA1060, the delivered BIP/BIPS may be replicated by providing the recipient’s Rep in trans. However, previous studies have shown no cross-reactivity between Rep proteins and DSOs from extensively homologous plasmids [29, 30]. Therefore, BIP/BIPS plasmids are not expected to replicate, even in *Bacillus* recipients with native plasmids belonging to the same family as pTA1060.

Rolling-Circle-Replicating (RCR) plasmids have tight regulatory mechanisms that control replication and copy number by preventing the Rep protein from catalyzing multiple rounds of DNA synthesis. The availability of Rep proteins is an important factor in the rate-limiting step of the rolling-circle plasmid process [30]. Although the replication mechanism for pTA1060 origin has not yet been elucidated, the availability of Rep proteins may also be important for the rolling-circle replication of BIP/BIPS. However, the initial construction of BIP using the native promoter of the *rep* gene for its expression resulted in considerably slow growth of *Bacillus* strains and difficulty in detecting plasmids. The results suggest that the native *rep* gene integrated into the chromosome may not sufficiently produce Rep proteins to maintain the integrative plasmids in the *Bacillus* donor. Moreover, the insufficient production of the Rep protein was overcome by replacing the native promoter of *rep* with a constitutive promoter, which remarkably increased the copy number of the BIP (Fig. 2B) and enabled the next step in plasmid-delivery into recipient cells. The results indicate that regulating the expression of the *rep* gene located on the chromosome can control the copy number of the plasmid lacking the *rep* gene. Therefore, BIP system may be widely used in studies that require plasmid copy number control.

As shown in Fig. 4, the rate of target gene deletion via BIP varied among the four tested *Bacillus* strains. The efficiency of homologous recombination can be affected by the size of homologous arms [12] and the distance between them [9]. However, the size and distance of the homologous arms for BIP/BIPS were similar for all tested strains. The different target gene deletion rates may be due to variability in the recombination efficiency across *Bacillus* species. A previous study supported these different rates of homologous recombination in bacteria [31]. Nevertheless, the BIPS system exhibited nearly 100% mutation efficiency in all four test strains, indicating that it can overcome the different recombination rates across the wild-type *Bacillus* strains. Moreover, because the ICE system has been demonstrated to deliver DNA to a wide range of bacteria [25], the BIP/BIPS systems using MICE could be used for a variety of microorganisms beyond the *Bacillus* species.

## Conclusion

Here, we developed *Bacillus* integrative plasmid systems, BIP and BIPS, using a broad-host-range plasmid-delivery system, MICE, to induce genomic mutations in wild-type *Bacillus* strains. The BIPS system exhibited a genomic mutation efficiency of nearly 100% in various wild-type *Bacillus* strains. Therefore, the BIP/BIPS systems can be powerful genetic tools for genomic mutagenesis in Cas9-sensitive or novel undomesticated *Bacillus* strains.

## Materials and methods

### Strains and culture conditions

All strains used in this study are listed in Additional file 1: Table S1. *E. coli* MC1061 and DH5α cells were used to construct recombinant plasmids. *E. coli*, donor strain (MICEaRep), and *B. velezensis* GB03 were cultured in LB (Difco, Thermo Fisher Scientific, Waltham, MA, USA) medium at 37 °C. *B. atrophaeus* and *B. mojavensis* cells were cultured in LB medium at 30 °C. *B. pumilus* cells were cultured on tryptic soy agar or broth (Difco) at 30 °C. When required, the medium was supplemented with ampicillin (100 µg/mL), chloramphenicol (15 µg/mL for *B. atrophaeus* and *B. pumilus*, 5 µg/mL for other *Bacillus* strains, and 50 µg/mL for in vivo recombination), neomycin (10 µg/mL), d-xylose (1%, w/v), and d-alanine (100 µg/mL) (Sigma-Aldrich, St. Louis, MO, USA).

### Construction of *Bacillus* conjugation donor and integrative plasmid

The plasmids and primers used in this study are listed in Additional file 1: Tables S2 and S3, respectively. pSGC2-aReptcR used to integrate the *rep* gene in the *amyE* locus of the *Bacillus* donor was constructed as follows: two DNA fragments corresponding to the upstream and downstream regions of *amyE* were amplified from the chromosome of *B. subtilis* 168 using the primers 168-amy-FF/168-amy-FR and 168-amy-BF2/168-amy-BR, respectively. The tetracycline resistance and *rep* genes were amplified from pBC16 [32] and pAD123 using GGC-tcR-F/GGC-tcR-R and pTA-rep-F2/pTA-rep-R2, respectively. All fragments were cloned into pSGC2 [33] using Golden-Gate assembly as previously reported [23] to construct pSGC2-aReptcR. The resulting plasmid was introduced into BS5918 to generate the MICEaRep.

The plasmid backbone for the BIP was modified from that of pSGC2 [33]. To replace *E. coli oriT* of pSGC2 with *Bacillus oriT* for MICE conjugation, the plasmid pA3D-gfpTi [24] was digested with HindIII and SacI, and the small fragment was ligated with HindIII- and SacI-digested pSGC2 to construct pSGC2i. The DSO region and neomycin resistance gene (*neo*) were amplified from pAD123 and pHCas9 [12], using pSGC2i-DSO-F2/DSO-R3 and 4iN-neo-F/pSGC2i-neo-R, respectively. The two PCR fragments and SacI- and NsiI-digested pSGC2i were fused to construct the plasmid pSGC2iN using a cold fusion cloning kit (System Biosciences. Palo Alto, CA, USA) according to the manufacturer’s instructions. The plasmid pSGC4iN containing a synthetic gene circuit was constructed as follows: to construct pAgR-Pxyl, SacI- and FseI-digested pAgR-Pgrac [23] was fused with *xylR*-P_*xylA*_ amplified from the chromosome of BS5417 [9] using xylR-xyl-F/xylR-xyl-R. Next, pAgR-Pxyl was digested with FseI and SpeI and fused with the *lacI* gene amplified from pAgR-Pgrac using lacI-F2/lacI-R to construct pAgR-Pxyl-lacI. pAgR-Pxyl-lacI was digested with SacI and XmaI, and the small fragment was ligated with SacI- and XmaI-digested pSGC2iN to construct the plasmid pSGC4iN.

To construct a BIP to delete target genes, two homologous arm fragments corresponding to upstream and downstream target genes were amplified from each target chromosome using the primers listed in Additional file 1: Table S3. The *cat* gene under P_*spac*_ was amplified from pA-xylR2 [9] using Pspac-F/cat-R. The homologous arms and P_*spac*_-*cat* fragments were cloned into pSGC2iN or pSGC4iN using the Golden Gate assembly. After introducing the resulting plasmid into MICEaRep as a donor strain, it was introduced to target recipients according to a previously reported conjugation method [24].

### Plasmid copy number determination by quantitative real-time PCR (qRT–PCR)

To compare the plasmid copy numbers between pAD123 and the *Bacillus* integration plasmid pSGC2iN under the same culture conditions, insertion of the *neo* gene into pAD123 was required. Accordingly, the pMGold-neoR plasmid with *neo* in the pAD123 backbone was constructed as follows: pMGold-sCBE4 [23] was digested with SacI and NsiI and a large fragment fused with the *neo* gene amplified from pHCas9 using sac-neoR-F/nis-neoR-R to construct pMGold-neoR. The copy numbers of pMGold-neoR in *B. subtilis* 168 and pSGC2iN in MICEaRep were determined. Total DNA was extracted from *Bacillus* strains using the AccuPrep^®^ Genomic DNA Extraction Kit (Bioneer Co., Daejeon, Republic of Korea) according to the manufacturer’s instructions. qRT-PCR was performed using iQ™ SYBR^®^ Green Supermix (Bio-Rad Laboratories, Hercules, CA, USA) as previously described [34]. The *dnaN* and *neo* genes were selected as single-copy reference genes on the chromosome and target gene on the plasmid, respectively. Specific primer sets, dnaN-F/dnaN-R, and neoR-F/neoR-R were used to amplify products of approximately 150 bp. The PCR mixtures (total volume, 20 µL) contained 10 µL iQ™ SYBR^®^ Green Supermix (2x), 1.0 µL forward and reverse primers (10 µM), and 1 µL diluted (10 − 2) sample DNA. The reaction conditions were as follows: 95 °C for 5 min; 45 cycles of 95 °C for 10 s, 55 °C for 10 s, and 72 °C for 30 s; followed by a gradient temperature from 55 to 95 °C. The plasmid copy number was calculated by dividing the mean of the *neo* gene in the plasmid by the mean of the *dnaN* gene. All experiments were performed in three independent biological replicates.

## Supplementary Information

**Additional file 1:** **Figure S1. **Map of pAD123 and sequence of DSO regions. The DSO region of pTA1061 origin is indicated by yellow shading. Inverted repeated sequences and the putative origin nick site within the sequence are indicated with arrows and a triangle, respectively. The -35, -10, and RBS sequences of the putative promoter of the *rep* gene are underlined. DSO, double-strand origin of replication. **Figure S2. **Confirmation of antibiotic resistance for 54 transconjugants of *B. pumilus* with integrative plasmid pSGC2iN-Pmapr. The single-crossover integration (SCO) and double-crossover integration (DCO) showed cm^R^/neo^R^ and cm^R^/neo^S^ phenotypes, respectively. Approximately 94.4% (51/54) of the total transconjugants showed a cm^R^/neo^R^ phenotype and were identified as SCO. The arrows indicate cm^R^/neo^S^ colonies. TSA, tryptic soy agar; cm, chloramphenicol; neo, neomycin; cm^R^, chloramphenicol resistant; neo^R^, neomycin resistant; neo^S^, neomycin sensitive. **Figure S3.** PCR analysis to identify the genotypes of the mutant strains. (**A**) The chromosome structure of wild-type strain (*B. pumilus*, *B. atrophaeus*, *B. mojavensis*, or *B. velezensis*) and the deletion mutants. The arrows indicate primer binding regions. (**B**) PCR analysis to confirm gene deletions, using primer sets offF/offR. “W” indicates a wild-type strain and “M” indicates a mutant strain. The expected sizes of the PCR products of *B. pumilus*, *B. atrophaeus*, *B. mojavensis*, and *B. velezensis* are 2.4 kb, 2.3 kb, 2.0 kb, and 2.6 kb for WT and 3.2 kb, 3.2 kb, 3.0 kb, and 3.3 kb for the mutant, respectively. **Table S1. **Bacterial strains used in this study. **Table S2. **Plasmids used in this study. **Table S3. **Primers used in this study. 

## Data Availability

All data generated or analyzed during this study are included in this published article and its Additional file 1. The sequences of pSGC2iN-Pmapr and pSGC4iN-Pmapr have been deposited in GenBank under Accession numbers OP889289 and OP889290, respectively.

## Abbreviations

CRISPR: Clustered regularly interspaced short palindromic repeats
CBE: Cytosine base editor
ICE: Integrative and conjugative element
MICE: Modified ICE
BIP: *Bacillus* integrative plasmid
DSO: Double strand origin of replication
BIPS: BIP with a synthetic gene circuit
qRT-PCR: Quantitative Real-time PCR
