# Supplementary material for: Bacillus integrative plasmid system combining a synthetic gene circuit for efficient genetic modifications of undomesticated Bacillus strains
Source: Microb Cell Fact. 2022 Dec 14;21:259. doi: 10.1186/s12934-022-01989-w (PMC9753358; doi:10.1186/s12934-022-01989-w)
Supplement: Supplementary file 1 — Additional file 1: Figure S1. Map of pAD123 and sequence of DSO regions. The DSO region of pTA1061 origin is indicated by yellow shading. Inverted repeated sequences and the putative origin nick site within the sequence are indicated with arrows and a triangle, respectively. The -35, -10, and RBS sequences of the putative promoter of the rep gene are underlined. DSO, double-strand origin of replication. Figure S2. Confirmation of antibiotic resistance for 54 transconjugants of B. pumilus with integrative plasmid pSGC2iN-Pmapr. The single-crossover integration (SCO) and double-crossover integration (DCO) showed cmR/neoR and cmR/neoS phenotypes, respectively. Approximately 94.4% (51/54) of the total transconjugants showed a cmR/neoR phenotype and were identified as SCO. The arrows indicate cmR/neoS colonies. TSA, tryptic soy agar; cm, chloramphenicol; neo, neomycin; cmR, chloramphenicol resistant; neoR, neomycin resistant; neoS, neomycin sensitive. Figure S3. PCR analysis to identify the genotypes of the mutant strains. (A) The chromosome structure of wild-type strain (B. pumilus, B. atrophaeus, B. mojavensis, or B. velezensis) and the deletion mutants. The arrows indicate primer binding regions. (B) PCR analysis to confirm gene deletions, using primer sets offF/offR. “W” indicates a wild-type strain and “M” indicates a mutant strain. The expected sizes of the PCR products of B. pumilus, B. atrophaeus, B. mojavensis, and B. velezensis are 2.4 kb, 2.3 kb, 2.0 kb, and 2.6 kb for WT and 3.2 kb, 3.2 kb, 3.0 kb, and 3.3 kb for the mutant, respectively. Table S1. Bacterial strains used in this study. Table S2. Plasmids used in this study. Table S3. Primers used in this study. [file 12934_2022_1989_MOESM1_ESM.docx]

***Bacillus* integrative plasmid system combining a synthetic gene circuit for efficient genetic modifications of undomesticated *Bacillus* strains**

**
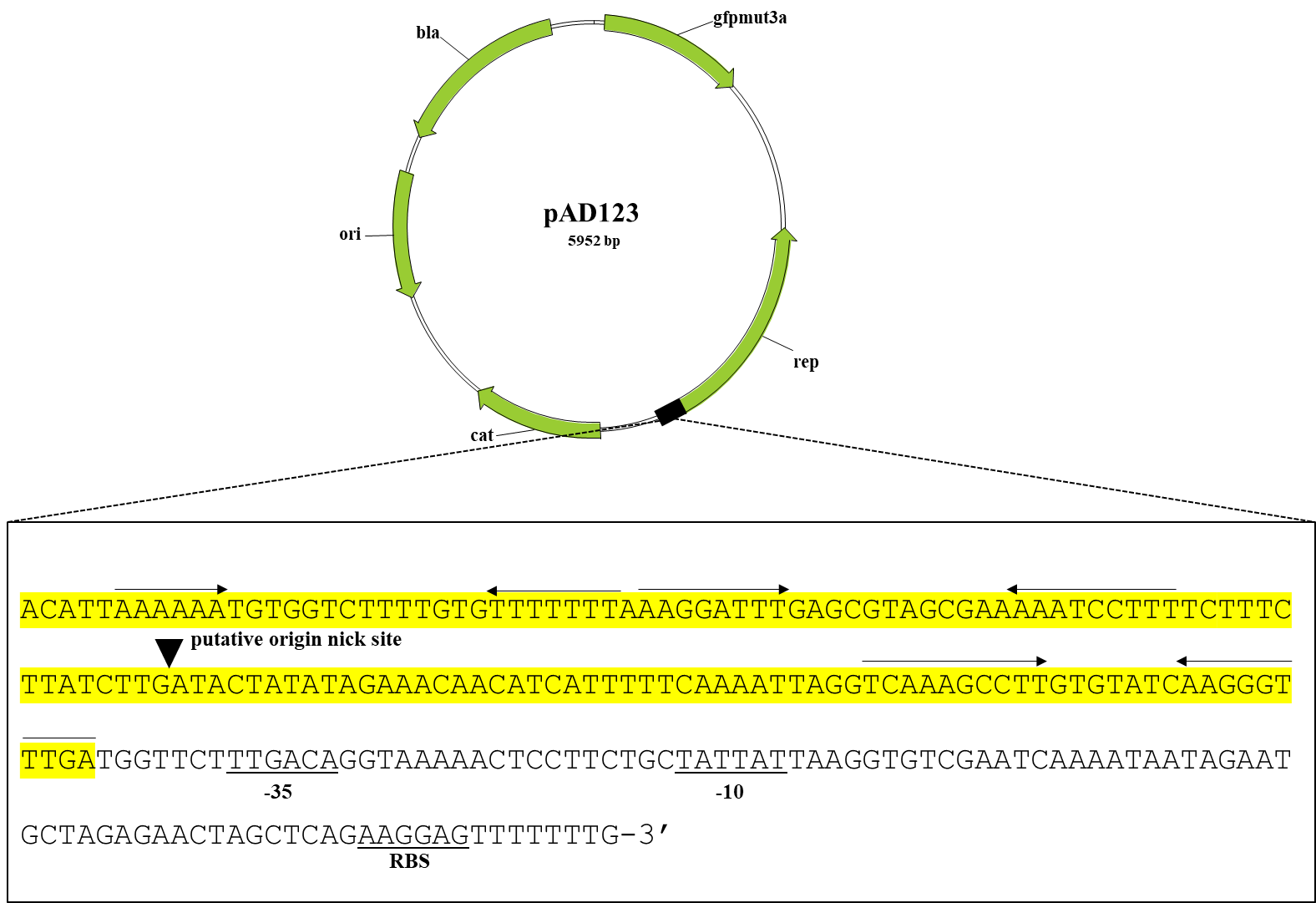
**

**Fig. S1** Map of pAD123 and sequence of DSO regions. The DSO region of pTA1061 origin is indicated by yellow shading. Inverted repeated sequences and the putative origin nick site within the sequence are indicated with arrows and a triangle, respectively. The -35, -10, and RBS sequences of the putative promoter of the *rep* gene are underlined. DSO, double-strand origin of replication.

**
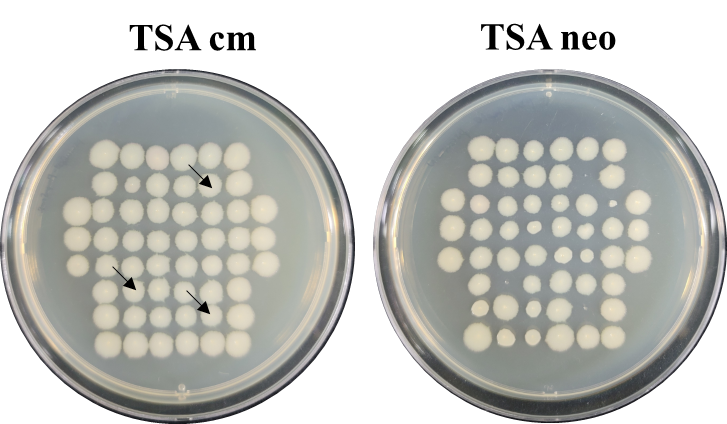
**

**Fig. S2** Confirmation of antibiotic resistance for 54 transconjugants of *B. pumilus* with integrative plasmid pSGC2iN-Pmapr. The single-crossover integration (SCO) and double-crossover integration (DCO) showed cm^R^/neo^R^ and cm^R^/neo^S^ phenotypes, respectively. Approximately 94.4% (51/54) of the total transconjugants showed a cm^R^/neo^R^ phenotype and were identified as SCO. The arrows indicate cm^R^/neo^S^ colonies. TSA, tryptic soy agar; cm, chloramphenicol; neo, neomycin; cm^R^, chloramphenicol resistant; neo^R^, neomycin resistant; neo^S^, neomycin sensitive.

**
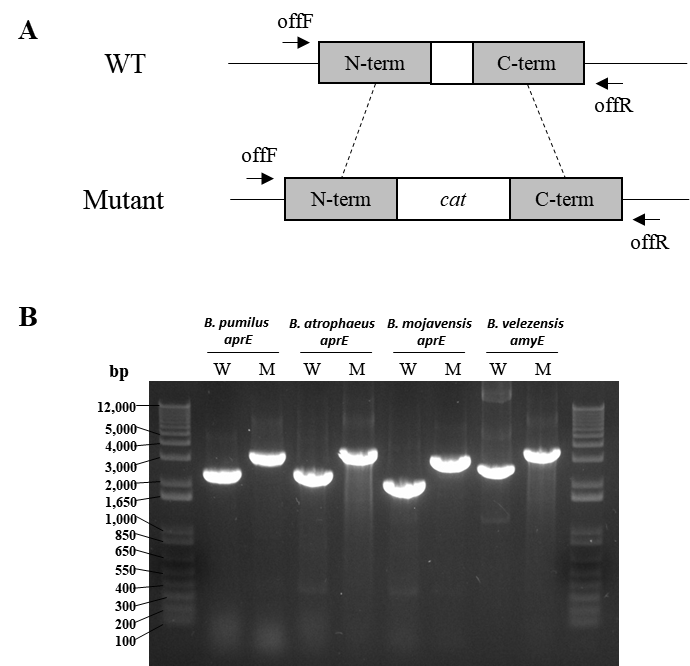
**

**Fig. S3** PCR analysis to identify the genotypes of the mutant strains. (**A**) The chromosome structure of wild-type strain (*B. pumilus*, *B. atrophaeus*, *B. mojavensis*, or *B. velezensis*) and the deletion mutants. The arrows indicate primer binding regions. (**B**) PCR analysis to confirm gene deletions, using primer sets offF/offR. “W” indicates a wild-type strain and “M” indicates a mutant strain. The expected sizes of the PCR products of *B. pumilus*, *B. atrophaeus*, *B. mojavensis*, and *B. velezensis* are 2.4 kb, 2.3 kb, 2.0 kb, and 2.6 kb for WT and 3.2 kb, 3.2 kb, 3.0 kb, and 3.3 kb for the mutant, respectively.

**Table S1.** Bacterial strains used in this study.

| **Strain** | **Genotype** | **Reference** |
| --- | --- | --- |
| ***Escherichia coli*** |  |  |
| DH5α | F′ Φ80*lacZ*△M15 △(*lacZYA*-*argF*)U169 *deoR* *recA1* *endA1* *hsdR17*(rk^-^, mk^+^) *phoA* *supE*44 *thi*-1 *gyrA*96 *relA1* | Enzynomics |
| MC1061 | *araD*139 △(*araA*-*leu*)7697 △(*lac*)X74 *galK*16 *galE*15(*GalS*) lambda- e14- *mcrA0* *relA1* *rpsL*150(strR) *spoT1* *mcrB1* hsdR2 | Laboratory stock |
| ***Bacillus*** |  |  |
| BS5918 | *B. subtilis* 168 △*rapI*-*attR*::*hyg*^R^ △*alrA*::Sp^R^-P*_xylA_*-*rapI* △*oriT*_ICE_ △*sigK* | [1] |
| MICERep | BS5918 △*amyE*::*rep* | This study |
| MICEaRep | BS5918 △*amyE*::P*_ara_*-*rep* | This study |
| *Bacillus pumilus^c^* | Wild type | KCTC 3348 |
| *Bacillus atrophaeus^c^* | Wild type | KCCM 41394 |
| *Bacillus mojavensis^c^* | Wild type | KCCM 42665 |
| *Bacillus velezensis* GB03 | Wild type | Laboratory stock |

**Table S2.** Plasmids used in this study.

| **Plasmid** | | **Description** | **Reference** |
| --- | --- | --- | --- |
| pAD123 | *E.coli* -*Bacillus* shuttle vector | | *Bacillus* Genetic Stock Center |
| pBC16 | Template for amplifying tetracycline resistance gene | | [2] |
| pHCas9 | Template for amplifying neomycin resistance gene | | [3] |
| pA-xylR2 | Template for amplifying P*_spac_*–chloramphenicol resistant gene (*cat*) fusion cassette | | [4] |
| pMGold-neoR | pAD123 derivative containing neomycin resistance gene | | This study |
| pSGC2 | pUC19 derivative containing Golden-Gate assembly site | | [5] |
| pSGC2i | pSGC2 derivative containing *oriT_ICE_* (BS168) | | This study |
| pSGC2iN | pSGC2i derivative containing neomycin resistance gene | | This study |
| pSGC4iN | pSGC2iN derivative containing synsthtic gene circuit | | This study |
| pAgR-Pgrac | Plasmid pAD123 derivative containing P*_grac_* | | [6] |
| pAgR-Pxyl | pAgR-Pgrac derivative replacing P*_grac_* with P*_xylA_* | | This study |
| pAgR-Pxyl-lacI | pAgR-Pxyl with *lacI* under P*_xylA_* | | This study |
| pSGC2-aReptcR | pSGC2 dereivative, generating P*_ara_*-*rep*-tc^R^ at *amyE* locus | | This study |
| pSGC2iN-Pmapr | pSGC4iN derivative, inserting P*_spac_*-*cat* at *aprE* locus of *B. pumilus* | | This study |
| pSGC4iN-Pmapr | pSGC4iN derivative, inserting P*_spac_*-*cat* at *aprE* locus of *B. pumilus* | | This study |
| pSGC4iN-Asapr | pSGC4iN derivative, inserting P*_spac_*-*cat* at *aprE* locus of *B. atrophaeus* | | This study |
| pSGC4iN-Mjapr | pSGC4iN derivative, inserting P*_spac_*-*cat* at *aprE* locus of *B. mojavensis* | | This study |
| pSGC4iN-GBamy | pSGC4iN derivative, inserting P*_spac_*-*cat* at *amyE* locus of *B. velezensis* GB03 | | This study |

**Table S3.** primers used in this study

| **Primer** | **Sequence (5’ to 3’)** | **Purpose** |
| --- | --- | --- |
| xylR-xyl-F | GAGACGATGCCAAAGAGCTCAGATCTTGATTAATTAATTCAGAACGCTCGG | Primers for construction of pAgR-Pxyl-lacI |
| xylR-xyl-R | AGACTAGTTTTTGGCCGGCCTTAAGTGAACAAGTTTATCCATC |  |
| lacI-F2 | TTGTTCACTTAAGGCCGGCCATCAAAGGGGGAAATGACAA |  |
| lacI-R | GATCCTTACTCGAGACTAGTTCACTGCCCGCTTTCCAGTC |  |
| pSGC2i-DSO-F2 | AGAAAGCCCAAAAGGAGCTCATTCCCGGGGCTTTAGTTGAAGAATAAAGACC | Primers for construction of the pSGC2iN |
| DSO-R3 | TCAAACCCTTGATACACAAG |  |
| 4iN-neo-F | CTTGTGTATCAAGGGTTTGATGATGACACAGAAGAAGGCG |  |
| pSGC2i-neo-R | GCAATTAATGTGAGATGCATCAATGCCGGGATAGACTGTA |  |
| 168-amy-FF | CTGGTCTCCATTGATGTTTGCAAAACGATTCAAAACC | Primers for construction of the pSGC2-aReptcR |
| 168-amy-FR | ACGGTCTCCCCTTCGATCAGACCAGTTTTTAATTTG |  |
| pTA-rep-F2 | CTGGTCTCCAAGGCGATAAAGATTGACAGTATAATAGTCAATTACTATAATAAAATTGGAGAACTAGCTCAGAAGGAG |  |
| pTA-rep-R2 | ACGGTCTCCGAGTGTGAATCGCTAAGAAACCAT |  |
| GGC-tcR-F | CTGGTCTCCACTCAGCAATCGCGCCCTTTAATG |  |
| GGC-tcR-R | ACGGTCTCCTCCTGTTGTATAAGTGATGAAATACTG |  |
| 168-amy-BF2 | CT GGTCTCCAGGATCGACATGGATGAGCGATGA |  |
| 168-amy-BR | ACGGTCTCCAAACTCAATGGGGAAGAGAACCGC |  |
| sac-neoR-F | AGGCGAGAGACGATGCCAAAGAGCTCTGATGACACAGAAGAAGGCG | Primers for construction of pMGold-neoR |
| nis-neoR-R | CAAGTTAAGGGATGCAGTTTATGCATCAATGCCGGGATAGACTGTA |  |
| dnaN-F | GCACTTGCCGCAGATTGA | Primers for quantitative real-time PCR |
| dnaN-R | AATGCAAGACGGTGGCTATC |  |
| neoR-F | CGGATATTGAGATGATGTGTGTCATGTC |  |
| neoR-R | GACCATGTGTAAGCGGCCAATC |  |
| 1G-PmaprE-FF | CTGGTCTCCATTGGAGTGAATTAGGTGAAACAAGTG | Primers for amplifying homologous arm fragments to delete target gene |
| 1G-PmaprE-FR | AC GGTCTCC CCTT ACATTTTGTCCAGCGTATCC |  |
| 3GX-PmaprE-BF2 | CTGGTCTCCACTCGGTAGATCGAGCATATGACAAG |  |
| 3GX-PmaprE-BR2 | ACGGTCTCCAAACTGAGACGGATGGAAAACGCA |  |
| 1G-asapr-FF | CTGGTCTCCATTGGATAGAGCTGGGCAAATCCA |  |
| 1G-asapr-FR | ACGGTCTCCCCTTTGACTGAGCTAATGCGTGAG |  |
| 3GX-asapr-BF | CTGGTCTCCACTCGAGCCAAACCCTTTCCAAGA |  |
| 3GX-asapr-BR | ACGGTCTCCAAACCGATACGTTTCAGGATTTAGC |  |
| 1G-mjapr-FF | CTGGTCTCCATTGGAATGTTCATCCCTTTTCCTTC |  |
| 1G-mjapr-FR | ACGGTCTCCCCTTGTGCAACATGATCTTCTTCC |  |
| 3GX-mjapr-BF | CTGGTCTCCACTCACAAACCCATACCAAGACGG |  |
| 3GX-mjapr-BR | ACGGTCTCCAAACCCGTTATCAGGAGAATAAGG |  |
| GB-amy-FF | CTGGTCTCCATTGTGCGGTCACTTTTGCGGTAG |  |
| GB-amy-FR | ACGGTCTCCCCTTAACGTTTCAGATAGGCCTGC |  |
| GB-amy-BF | CTGGTCTCCACTCTTTAGGCTGGGCAGTGATTG |  |
| GB-amy-BR | ACGGTCTCCAAACCATTAATGCGGAAGATAACCATTC |  |
| GGC-cat-F | CTGGTCTCCAAGGGAAAAGGATTTTTCGCTACGC | Primers for amplifying *cat* |
| Pspac-F | CTGGTCTCCAAGGTACACAGCCCAGTCCAGACT |  |
| cat-R | ACGGTCTCCGAGTGCGAATGGCGACTAACGGGG |  |
| PmaprE-offF | GTTCTTGTGACAGAGGCAGG | Primers for identifying genotype of the mutant strain |
| PmaprE-offR | GCGCTAAAATCATCAGTAGAAC |  |
| asapr-offF | GCTCATATAAACTTCTTGCAGC |  |
| asapr-offR | TTCCTTTGCTGCTTCTGCTG |  |
| mjapr-offF | CTCTTTATTTTCCAGGCGTG |  |
| mjapr-offR | GTTATTGCTTATCGTGTCTG |  |
| GB-amy-offF | AGACACCAAAGAAGAACTGC |  |
| GB-amy-offR | GGTGATGGTTTCCAGATTGTTG |  |

Underlined sequences are the restriction enzyme sites.

**References**

1. Jeong D-E, Kim MS, Kim H-R, Choi S-K. Cell factory engineering of undomesticated *Bacillus* strains using a modified integrative and conjugative element for efficient plasmid delivery. Front Microbiol. 2022**:**176.

2. Palva A, Vigren G, Simonen M, Rintala H, Laamanen Pi. Nucleotide sequence of the tetracycline resistance gene of pBC16 from *Bacillus cereus*. Nucleic Acids Res. 1990; 18**:**1635.

3. So Y, Park S-Y, Park E-H, Park S-H, Kim E-J, Pan J-G, et al. A highly efficient CRISPR-Cas9-mediated large genomic deletion in *Bacillus subtilis*. Front Microbiol. 2017; 8**:**1167.

4. Jeong D-E, Park S-H, Pan J-G, Kim E-J, Choi S-K. Genome engineering using a synthetic gene circuit in *Bacillus subtilis*. Nucleic Acids Res. 2015; 43**:**e42.

5. Riu M, Kim MS, Choi SK, Oh SK, Ryu CM. Elicitation of Innate Immunity by a Bacterial Volatile 2-Nonanone at Levels below Detection Limit in Tomato Rhizosphere. Mol Cells. 2022; 45**:**502-511.

6. Kim MS, Kim H-R, Jeong D-E, Choi S-K. Cytosine Base Editor-Mediated Multiplex Genome Editing to Accelerate Discovery of Novel Antibiotics in *Bacillus subtilis* and *Paenibacillus polymyxa*. Front Microbiol. 2021; 12.
